# Chloroform in Strychnia-Poisoning

**Published:** 1874-11

**Authors:** 


*CHICAGO MEDICAL EXAMINER—September*, 1874.

Chloroform in Strychnine Poisoning (*The New York Medical*

*Record, July* 1, 1874.)—A man took five grains of strychnine, with a suicidal intent. He was given twenty grains of sulphate of zinc, which produced vomiting. Convulsions had occurred repeatedly, however, and he was seized with one of tetanic form at the time of coming under observation. Every muscle was rigid, and tetanus was complete. Opisthotonos, irregularity of the pulse, varying from 120 to 140 in the minute, with all the accompanying symptoms, were noticeable.

He was immediately placed under the influence of chloroform. The convulsions ceased from the commencement of the anæsthesia, under which the patient was fully kept for three hours. The chloroform was then removed, but the patient did not awake until six hours afterwards—a case of recovery.

